# Failure to define level 1 care

**DOI:** 10.1186/cc9902

**Published:** 2011-03-11

**Authors:** B Fletcher, C Dames, S Hutchinson, S Fletcher

**Affiliations:** 1 Norfolk & Norwich Hospital, Norwich, UK

## Introduction

This observational prevalence study applies three definitions of level 1 care to a hospital-wide cohort of adult patients in a university hospital and a district general hospital, to test the validity of measures to define at-risk patients outwith critical care. This report provides a first look at the university arm of the study. The importance of correctly applying an acceptable definition is twofold. Firstly, an individual at risk of deterioration may be highlighted to critical care outreach services (CCOS). Secondly, a population of at-risk patients may identify an unmet resource need. The three common definitions are: Intensive Care Society (ICS) [1], Department of Health: Comprehensive Critical Care (CCC) [2] and Association of UK University Hospitals (AUKUH) [3]. The earliest definition of a level 1 patient by CCC identifies recent critical care discharges and/or deteriorating patients needing CCOS [1]. The ICS definition adds detail to this by scoping the options for monitoring or clinical intervention [2]. The AUKUH identifies two subgroups within level 1: acutely ill or deteriorating patients, and stable patients with greater nursing dependency [3].

## Methods

Data were collected from all inpatients by a team of trained researchers, using hand-held computers, over 5 days. The paediatric, maternity, oncology and emergency units were excluded. A central data controller guarded against omissions or duplications. The acuity criteria dataset was constructed by a regional expert critical care steering committee. The dataset from the university and district hospital sites have not yet been combined.

## Results

A total of 696 patients were included, representing >97% of patients in surveyed wards. Within the 24-hour period before data collection: four patients had CCOS review, nine had stepped down from level 2/3 care and 51 had MEWS >3. In total, 371 patients (53%) met the criteria of at least one of the definitions, if not all three.

## Conclusions

A significant proportion of adult patients meet one or more of the current definitions for level 1. We suggest that the current definitions may be unhelpful in identifying at-risk patients outside critical care. Further work is planned to investigate whether certain criteria, or combinations thereof, are better predictors of unmet clinical need, or contribute more to patient safety.
